# Protocol for the Evaluation of Data-Related Processes and Challenges at Level 1–4 Trauma Centers in Arkansas: A Mixed-Methods Case Study

**DOI:** 10.21203/rs.3.rs-3477512/v1

**Published:** 2023-10-30

**Authors:** Cara L. Conner, Mersady C. Redding, Emel Seker, Melody L. Greer, Maryam Y. Garza

**Affiliations:** University of Arkansas for Medical Sciences; University of Arkansas for Medical Sciences; University of Arkansas for Medical Sciences; University of Arkansas for Medical Sciences; University of Arkansas for Medical Sciences

**Keywords:** registries, Arkansas Trauma Registry, qualitative research, thematic analysis, real-world data

## Abstract

**Background:**

In the following manuscript, we describe the detailed protocol for a mixed-methods, observational case study conducted to identify and evaluate existing data-related processes and challenges currently faced by trauma centers in a rural state. The data will be utilized to assess the impact of these challenges on registry data collection.

**Methods:**

The study relies on a series of interviews and observations to collect data from trauma registry staff at level 1–4 trauma centers across the state of Arkansas. A think-aloud protocol will be used to facilitate observations as a means to gather keystroke-level modeling data and insight into site processes and workflows for collecting and submitting data to the Arkansas Trauma Registry. Informal, semi-structured interviews will follow the observation period to assess the participant’s perspective on current processes, potential barriers to data collection or submission to the registry, and recommendations for improvement. Each session will be recorded and de-identified transcripts and session notes will be used for analysis. Keystroke level modeling data derived from observations will be extracted and analyzed quantitatively to determine time spent performing end-to-end registry-related activities. Qualitative data from interviews will be reviewed and coded by 2 independent reviewers following a thematic analysis methodology. Each set of codes will then be adjudicated by the reviewers using a consensus-driven approach to extrapolate the final set of themes.

**Discussion:**

We will utilize a mixed methods approach to understand existing processes and barriers to data collection for the Arkansas Trauma Registry. Anticipated results will provide a baseline measure of the data collection and submission processes at various trauma centers across the state. We aim to assess strengths and limitations of existing processes and identify existing barriers to interoperability. These results will provide first-hand knowledge on existing practices for the trauma registry use case and will provide quantifiable data that can be utilized in future research to measure outcomes of future process improvement efforts. The potential implications of this study can form the basis for identifying potential solutions for streamlining data collection, exchange, and utilization of trauma registry data for clinical practice, public health, and clinical and translational research.

## Background

Registries play a critical role in clinical practice, research, and public health by providing a rich source of healthcare information that can be leveraged to improve patient and population health outcomes (1). The data collected to support registries are derived from local, state, and national sources, providing a window into healthcare at all levels. As more federal regulations require reporting to clinical data registries, the role of registries in clinical practice, research, and public health has never been more significant. In addition to being a rich data source for clinical research and public health surveillance, these specialized data sets are often relied on for use in routine clinical practice, which require data to be timely and of high quality.

A trauma registry is a data registry or repository with the explicit purpose of data collection of uniform, clinical data elements that “document acute care delivered to patients hospitalised with injuries” (2). In general, registry data can be used to support clinical practice (e.g., care coordination, performance improvement), clinical and public health research, and public health initiatives (e.g., surveillance, epidemiological and injury prevention) (3). Trauma registries are designed to “improve the efficiency and quality of trauma care” (2) and, at regional and national levels, trauma registries can offer large datasets that can be used for comprehensive public health surveillance of injuries (4) and to evaluate patient outcomes within and across facilities (2).

Trauma centers across the US are identified and designated by the American Trauma Society (5) into 5 different levels (levels 1–5), after completing designation and verification processes. Level determination is dependent on the kind of resources available at the center and the number of patients admitted yearly (6). This provides a national standard for trauma care in hospitals. Level 1 refers to a comprehensive regional resource that is a tertiary care facility central to the trauma system (e.g., Cedars–Sinai Medical Center, Massachusetts General Hospital) (5). In contrast, level 5 trauma centers provide only the initial evaluation and stabilization, have limited diagnostic capabilities, and most often prepare patients for transfer to higher levels of care (5).

### Arkansas Trauma Registry

The Arkansas Trauma Registry (ATR) (3) is a statewide trauma data collection and evaluation system composed of standardized data elements that include details of each injury event, patient demographics, prehospital information, diagnoses, in-hospital care, and eventual outcomes of the injured patients admitted to facilities across the rural state of Arkansas. The goal of the ATR (and registries in general) is to obtain, code, and maintain the data for analysis and eventual reporting of individual and aggregate results. Arkansas has level 1–4 trauma centers^[1]^, all of which participate in the ATR by providing a standardized set of real-world data^[2]^ from the electronic health record (EHR) on various trauma patients (referred to as cases). Sites across the state of Arkansas also participate in the National Trauma Data Bank^®^ (NTDB^®^) Trauma Program, which is the largest aggregation of US trauma registry data available for research and reporting (e.g., benchmarking and performance improvement) (7).

Sites participating in the ATR (and/or NTDB^®^) are required to submit data on an ongoing basis and invest a substantial amount of time and effort (not to mention, money) on collecting, coding, performing quality reviews of, and submitting data. In most cases, these processes – which are not unique to trauma registries – are highly manual and burdensome (2,4,8). Registries often rely on manual medical record abstraction (or chart review) to gather data from EHRs and emergency medical services (EMS) systems. Such manual approaches are expensive and, often, inefficient, primarily due to the complexities associated with data collection or abstraction and mapping of the data to fit the registry data model (9).

### Data Quality and Process Improvement

Medical information is complex. Designing a data collection process that captures its intricacies while smoothly fitting into the workflow is especially important. Because trauma registrars, registry coordinators, and program managers are directly involved in the data collection process, they have a good understanding of the procedures, workflows, and variables contributing to the overall quality of data that is submitted; and data collection can be improved with their guidance. Trauma registries need data quality improvements in multiple areas; most notably, standardization and missing data are common areas of weakness impacted by the collection process. A recent study found that 70% of trauma centers were missing comorbidity data (10). Historically, data quality monitoring and management have been inconsistent, but studies show that confidence in the registry data is directly related to data quality (11). In fact, periodic data quality reviews should be conducted using systematic processes to track and improve these issues (12). In general, data standardization must underlie the process of data acquisition and exchange to support generalized trauma care quality improvements. Curtis and colleagues (13) report that a standardized approach to reviewing trauma cases across hospitals is a priority for trauma quality improvement. Advances in systems interoperability, i.e., through the use of standards-based methods and direct EHR data exchange, have the potential to address these issues by automating part or all of the data collection and submission processes.

In the following manuscript, we describe the detailed protocol for a mixed-methods, observational case study conducted by researchers at the University of Arkansas for Medical Sciences (UAMS). This study incorporates the perspectives of experienced trauma personnel to identify aspects of registry submission that could be made more efficient. The results of this research has the potential to save money and enhance the quality of the data obtained, leading to improved performance and trauma standards of care, and, ultimately, better patient outcomes.

## Methods

### Objective

The primary objective of this study is to evaluate existing data collection and exchange processes of trauma centers in a rural state (Arkansas) to identify data-related challenges and the potential impact on registry data collection and submission to a state-based clinical data registry (Arkansas Trauma Registry). A secondary objective is to calculate the average time spent on data collection and submission by generating workflow and process-level metrics using keystroke-level modeling (KLM) (14).

### Study Design

This study employs a mixed-methods, observational case study design for data collection and analysis through the use of semi-structured observations and interviews (Table 1). A detailed investigation will be conducted of the current data collection processes for the ATR across level 1–4 trauma centers within the state of Arkansas. We will rely on direct observations and interviews as our primary data sources to (1) gather qualitative information from registry personnel across unique sites on current processes, challenges, and perceptions (primary objective) of the end-to-end data-related practices carried out at each site; (2) generate data- and workflow diagrams corresponding to the processes outlined by the site (primary objective); and (3) collect KLM data (quantitative) based on observations and verbal walkthroughs (secondary objective) using a think-aloud protocol. This study has received a determination of not human subjects research as defined in 45 CFR 46.102 by the UAMS Institutional Review Board.

### Study Participants

ATR personnel employed at level 1–4 trauma centers across the state of Arkansas will be recruited to participate in this study. Participants will be included if they are clinical, research, or data registry personnel who actively participate in the day-to-day data collection and submission processes for the ATR. More specifically, we are interested in the perspectives of trauma program managers or supervisors, registrars, coordinators, and data entry staff (all roles hereinafter referred to as registrars) responsible for capturing, entering, tracking, and submitting patient data to the ATR. There are no exclusion criteria for this study.

We will rely on a combination of purposive (15) and snowball (16, 17) sampling to identify and recruit participants from Arkansas trauma centers throughout the state. Purposive sampling is a commonly used sampling method in qualitative research (15) to select “information-rich cases for the most effective use of limited resources.” We will first identify trauma centers in Arkansas through publicly available information from the Arkansas Department of Health. Once sites have been identified, we will identify key personnel from each site for inclusion, engaging with the Arkansas Department of Health for assistance, where appropriate.

Participation is voluntary and informed consent to participate will be obtained. Emails will be sent to potential participants briefly outlining the objectives of the study and details of participation. In addition, contact information for the primary investigator (MYG) will be included to allow participants to follow up with questions or request additional information before committing to participate in the study. Those expressing interest will be scheduled for an observation and an interview session. In situations where a potential participant does not respond to the initial email, 3 additional follow-up emails will be attempted before ceasing recruitment attempts. Participation is completely voluntary. By participating in the observations and interviews, the participants are acknowledging informed consent, and the responses to questions and discussion points during each session will be considered part of the dataset collected for the study and will be included in the analysis.

At minimum, we aim to include personnel from at least 4 sites, each site representing a different trauma certification level. This will ensure we are able to identify potential differences between sites of different levels. From each site, we aim to include at least 1 person but will attempt to interview all that are available and willing to participate. It is likely that sites will vary in the number of relevant personnel, assuming anywhere between 1 and 5 personnel per site. Therefore, we look to recruit between 4 (1 person per site across 4 sites) to 20 (5 persons per site across 4 sites) total participants. However, recruitment will continue only until we have reached data saturation (18). In other words, as long as we reach our threshold of 1 person across 4 unique sites of varying trauma levels, we will stop recruitment if we establish that data collected during observations and interviews is repetitive and no new data is being expressed.

#### Compensation.

Participants will be eligible for compensation upon completion of study activities (one observation and one interview session). Participants will be eligible to receive a $50 gift card for completing one, 30-minute observation session, and another $50 gift card for completing one, 30-minute interview session, for a maximum of $100.

#### Risks and Benefits.

This research is an observational study that relies on interviews, observations, and surveys. The probability and magnitude of harm or discomfort anticipated in the research are not greater than those ordinarily encountered in daily life or during the performance of routine physical and psychological examinations or tests and where confidentiality is properly protected. There are no physical risks associated with this study. However, there is a potential loss of confidentiality risk for participants. Measures to protect the confidentiality of study data will be implemented, and every effort will be made to keep their information confidential; however, this cannot be guaranteed. For this reason, participants will be advised of this limitation during the informed consent process. Other than the compensation for participation, there is no direct benefit for participation in this study. However, knowledge gained from the study could potentially benefit patients in the future. We hope that the information gained will increase our knowledge of which sites and clinical studies are most appropriate for standards-based, data exchange technologies.

### Study Setting

This study will be conducted online (virtually) using the Zoom Video Conferencing platform (https://zoom.us/) licensed by UAMS. Virtual sessions will be closed and available by invitation only. Observations and interviews will be recorded (video and audio) for analysis. Registry personnel can join the virtual meeting room and opt-in to share their camera and computer screen(s) to display any relevant information during the session. All research activities will be conducted by appropriately qualified members of the research team in a discrete setting. Trained members of the research team will carry out the virtual sessions in a private room, such as a work or home office with a closed door and away from others, to maintain a private environment. Participants can join from their typical work environment (e.g., work or home office setting) and have full control over the setting from which they join. However, for the observations in particular, it is optimal that the participant joins from their primary work environment to facilitate accurate workflow with on-site technologies, including program layout and keystroke maneuvers.

We will allocate 30 minutes each for observation and interview sessions, for a total time commitment of 60 minutes. To accommodate participant schedules, the sessions can occur concurrently, as a single, 60-minute meeting, or be scheduled separately, as two distinct 30-minute sessions. In the event that the allocated time is not sufficient to complete either session, the study team will invite the participant to one additional 30-minute session to complete any remaining components of the observation and/or interview.

Observations and interviews will follow a semi-structured format, referencing an interview guide developed prior to study start. The observation/interview guide(s) will include a script with example prompts for guidance to help drive the conversation and encourage the interviewee to describe processes and opinions in as much detail as possible. The interview guide will be a working (or living) document that can be modified or adapted after each interview, to allow the research team to learn and gather new insight that will inform future interviews.

Observations and interviews will be proctored by a trained member of the research team who will be responsible for managing the meeting, relying on the interview guide for guidance. Responsibilities will include managing and maintaining the recording during the session, following up with prompts or additional, clarifying questions (where appropriate), capturing field notes (as needed), and closing out the session, which may or may not require scheduling a follow-up session. A second member of the research team may join the session as a technical backup and to assist with note-taking.

### Data Collection

**Figure 1:**
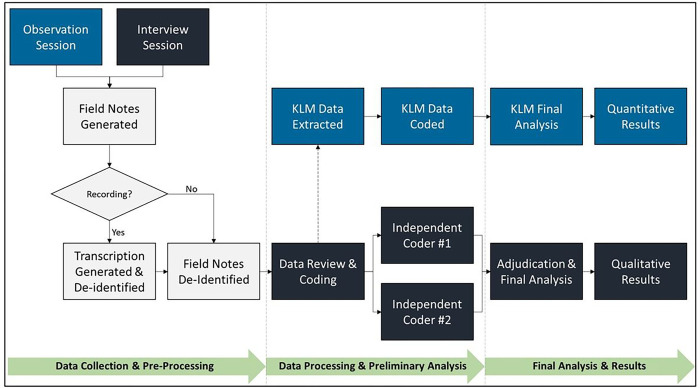
Methodology for Data Collection and Analysis KLM: Keystroke-level modeling

We will use semi-structured observations and interviews to collect data from personnel at Arkansas trauma centers that are involved in the end-to-end data collection and submission to the ATR. Figure 1 provides an overview of the data collection processes to be followed for this study.

Observations provide an avenue for participants to walk through current site-level processes with the research team. The walkthroughs follow a think-aloud protocol (19, 20), which will be used to allow the evaluator to assess the ease, challenges, and comprehension of the tasks being performed by the end-users (e.g., registrars collecting and submitting data to the ATR). Think-aloud protocols allow the interviewee to walk through the entire process from beginning to end, while verbally explaining steps aloud as they complete each task. Think-aloud facilitates a thorough description of existing processes without introducing potential biases of the researcher. For example, a participant describing how to exit an internet browser window may say, “I have completed my task and am ready to close out of the system. To do so, I am placing my hand on the mouse, moving the cursor to the right-hand corner of the browser window, where I notice a red ‘X’, and then, I single-click the red ‘X’ to close the window.” Each step will be analyzed and quantitative data extracted for the KLM analysis. Referring back to the example stated above, we can derive key operators and predict execution times as follows: the mental preparation and acknowledgement of a needed action (“ready to close out of the system”), assigned operator “M”, would take about 1.35 seconds; placing the hand on the mouse, operator “H”, would take about 0.40 seconds; movement of the mouse and pointing to the target, operator “P”, would take about 1.10 seconds; and the final “single click on the red ‘X’ to close the window”, operator “K”, would take about 0.20 seconds. These times would then be summed to get the overall time for the activity (t = 3.05 seconds).

In addition to generating the KLM results, data gathered during the course of the observation will be used to create data- and workflow diagrams, which will be reviewed by the interviewee(s) to ensure a common understanding of existing processes. Dataflow diagrams will be generated to depict the end-to-end movement of the data from the moment a trauma case is first identified, to when the final set of data for a case is submitted to the registry. Workflow diagrams will be generated to complement dataflow, and will depict how the site staff moves through the data-related processes from start to finish.

The semi-structured interviews, proctored by a trained member of the research team, are informal and conversational. Although the research team has developed several general questions as prompts for discussion, participants may express concerns or obstacles that are not adequately addressed by the standard prompts, so interviewers may expand with open-ended follow-up questions, as appropriate. Encouraging the interviewee to guide discussion creates flexibility during the interview that ensures attention to and appreciation for critical and unique processes at each site. In general, interviews are centered on the day-to-day activities and data-related tasks of each interviewee as it relates to their work with the ATR. Specific topics of interest include: standard process/work flow; typical work/case load on a daily, weekly, and/or monthly basis; typical data collection procedures; perceived issues or barriers to collection; perceived overall data quality; recommendations or suggestions for process improvement. No personal information will be collected.

### Data Analysis

**Figure 2:**
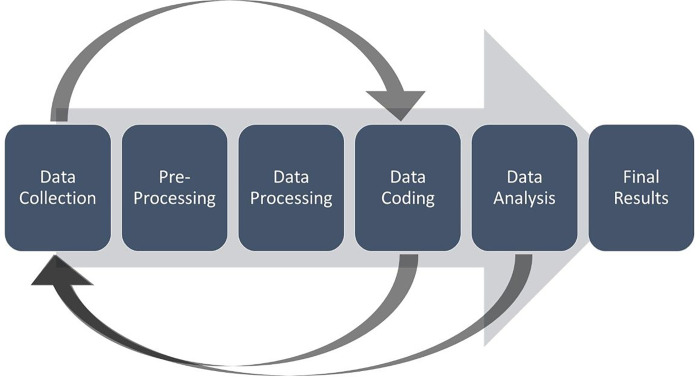
Flow of Data Collection and Analysis of Qualitative Data

Qualitative data from the observations and interviews will be analyzed using a constant comparative approach (21), which focuses on illuminating barriers and facilitators in technological, procedural, and personnel attributes as it relates to current data collection procedures. Figure 2 depicts the process flow from data collection to final analysis and results.

**Figure 3:**
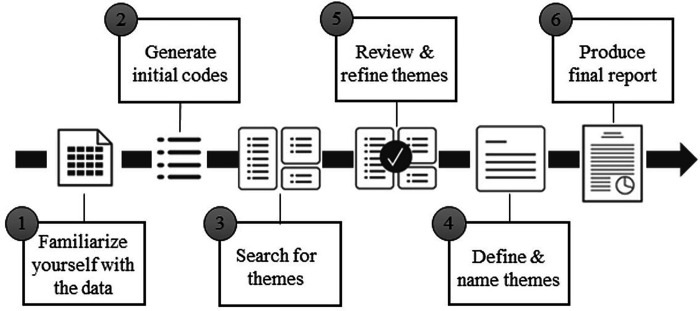
Six Phases of Thematic Analysis *Adapted from CJ Eubanks Fleming, Qualitative Methods for the Qualitatively Inclined* (25).

Through thematic analysis (Fig. 3), we aim to identify common themes related to processes, barriers, and time metrics. Using a Rapid Qualitative Evaluation approach (22–24), all qualitative data will be coded, independently, by two trained members of the research team. Upon completion, the coders will review their results and perform an adjudication of the coding using a consensus-driven approach. The full research team will be consulted in the event the coders cannot reach a consensus. We will supplement this data using the KLM process metrics and the data- and workflow diagrams, which will be used to calculate the average time spent on data collection.

### Data Management

Name and email of the participants will be used for the purposes of recruitment and communication only (i.e., to schedule and carry out observations and interviews sessions). This information will not persist with the actual study data and will not be included in the analysis; so there will not be a way to tie the study data back to the participant. Data collected through this study will not include any personal or identifiable information on any of the participants.

The sessions will rely on both audio and visual recordings to capture the activities and responses of the participants during the observations and interviews. We will use secure, virtual communications tools provided through and approved by UAMS to conduct the virtual observation and interview sessions. The recordings will be maintained on a secure UAMS-maintained server, tied to the Principal Investigator’s institutional web conferencing platform account (e.g., UAMS Zoom account, or similar). For added security, the recordings will be transferred from the web conferencing account to a secure, UAMS Box folder for more permanent storage, and removed from the web conferencing platform immediately following the session. Only the study team will have access to the study folders in UAMS Box. UAMS Box is approved by UAMS Information Security and the IRB as an acceptable tool for storing research data at the institution.

Written transcripts and research notes (paper and/or electronic) will be generated during each session and stored with the corresponding video recording for each session. Should paper documentation be used, the paper notes will be immediately transcribed into electronic format for storage with the full study dataset, and the paper transcripts will be shredded. Written transcripts will only include data relevant to the research project and will not include any identifiable information. Examples of items that would be contained within a transcription could include: a code to help distinguish subject responses (e.g., Subject A vs. B); the type of subject (e.g., registrar vs. trauma program manager); the date/time the interview occurred; and the observation/interview number.

Only IRB-approved members of the study team will have access to the data. User access to the Box folders will be carefully managed by the Principal Investigator. At the end of the study, all records will continue to be kept in a secure location for as long a period as dictated by the sponsor or the UAMS IRB and institutional policies.

## Discussion

This manuscript describes a mixed-methods, observational case study design for the evaluation of data-related processes and challenges at level 1–4 trauma centers in Arkansas. This study is designed to identify critical gaps in our understanding of the way in which data is acquired and exchanged for registry consumption and use. A potential implication of this study is that areas of improvement to the ATR’s data submission process are identified and reported. This information could form the basis for identifying potential solutions for streamlining data collection, exchange, and utilization in translational research on trauma registry data input.

As the saying goes, you cannot improve what you cannot measure. The results of this work will provide a baseline measure of the ATR data collection processes and the average time spent on data collection and submission at various trauma centers across the state. In rural areas like Arkansas, trauma personnel may serve in multiple roles at once in order to provide care to trauma patients in the emergency department, document trauma data, input it into the registry, and assess for performance improvement opportunities. This research is especially pertinent for trauma centers with limited resources that could benefit from increasing efficiency in the data collection and registry input process.

Case studies are often used in exploratory research to help illustrate the current attributes of a given phenomenon (26). In this case, we aim to obtain an in-depth understanding of current data collection practices at level 1–4 trauma centers submitting data to the ATR in order to assess strengths and limitations of existing processes and identify existing barriers to interoperability. The results from the observations and interviews will provide first-hand knowledge on existing practices for the ATR/trauma registry use case; and the KLM process will provide quantifiable data that can be utilized in future research to measure outcomes of future process improvement efforts. This new knowledge can support the identification of solutions for streamlining data collection, exchange, and utilization of trauma registry data for clinical practice, public health, and clinical and translational research.

## Figures and Tables

**Table 1:** Protocol Overview

**Context:** Level 1–4 trauma centers within the state of Arkansas.
**Objective:** To evaluate existing data-related processes and challenges currently faced by Arkansas trauma centers submitting data to the ATR.
**Study Design:** Mixed-Methods, Observational Case Study
**Data Collection:** A 30-minute observation period detailing walkthrough of typical data collection and submission to the ATR (to collect quantitative, KLM data), followed by a 30-minute informal interview describing challenges to data collection and submission to the ATR (to collect qualitative data).
**Analysis:** Thematic analysis of interview data (qualitative) to identify common themes as to the perceptions of current data-centric processes at sites. KLM of observation data (quantitative) to generate baseline time estimates for data collection and submission at each site.

ATR: Arkansas Trauma Registry; KLM: Keystroke-level Modeling

## Data Availability

Not Applicable.

## Footnotes

1 For a list of designated trauma centers in Arkansas, refer to the Arkansas Department of Health website: https://www.healthy.arkansas.gov/images/uploads/pdf/Designated_Trauma_Centers.pdf.

2 NIH Definition for Real-World Data (RWD): *refers to data relating to patient health status and/or the delivery of health care routinely collected provided outside of a research setting. RWD may be collected from a variety of sources including the patient, clinician, registries, or electronic medical records.*

## Abbreviations

ATR: Arkansas Trauma Registry
EHR: Electronic Health Record
NTDB^®^: National Trauma Data Bank
EMS: Emergency Medical Services
UAMS: University of Arkansas for Medical Sciences
KLM: Keystroke Level Modeling
IRB: Institutional Review Board

## References

[1] Gliklich RE, Dreyer NA, Leavy MB, Christian JB, editors. 21st Century Patient Registries: Registries for Evaluating Patient Outcomes: A User’s Guide: 3rd Edition, Addendum [Internet]. Rockville (MD): Agency for Healthcare Research and Quality (US); 2018 [cited 2022 May 20]. (AHRQ Methods for Effective Health Care). Available from: http://www.ncbi.nlm.nih.gov/books/NBK493818/29708678

[2] Moore L, Clark DE. The value of trauma registries. Injury. 2008 Jun;39(6):686–95. 18511052 10.1016/j.injury.2008.02.023

[3] Arkansas Department of Health. Arkansas Trauma Registry (ATR) [Internet]. 2017. Available from: https://www.healthy.arkansas.gov/programs-services/topics/arkansas-trauma-registry-atr

[4] Pollock DA. Trauma Registries and Public Health Surveillance Injuries [Internet]. Centers for Disease Control and Prevention: National Center for Health Statistics; p. 1–6. Available from: https://www.cdc.gov/nchs/data/ice/ice95v1/c11.pdf

[5] American Trauma Society. Trauma Center Levels Explained [Internet]. [cited 2023 Sep 29]. Available from: https://www.amtrauma.org/page/traumalevels

[6] ESO Solutions, Inc. Explainer: The Five Trauma Center Levels [Internet]. 2021 [cited 2023 Sep 29]. Available from: https://www.eso.com/blog/explainer-the-five-trauma-center-levels/

[7] American College of Surgeons. ACS. [cited 2023 Jun 29]. National Trauma Data Bank. Available from: https://www.facs.org/quality-programs/trauma/quality/national-trauma-data-bank/

[8] What is a Trauma Registry? [Internet]. ESO. 2020 [cited 2021 Mar 1]. Available from: https://www.eso.com/blog/what-is-a-trauma-registry/

[9] Blumenthal S. Improving Interoperability between Registries and EHRs. AMIA Summits Transl Sci Proc. 2018 May 18;2018:20–5. PMC5961768 29888033

[10] Fransman R, Kent AJ, Haut ER, Kar AR, Sakran JV, Stevens K, Facility disparities in reporting comorbidities to the National Trauma Data Bank. Am J Surg. 2018;216(3):401–6. 29395020 10.1016/j.amjsurg.2018.01.031

[11] O’Reilly GM, Gabbe B, Moore L, Cameron PA. Classifying, measuring and improving the quality of data in trauma registries: A review of the literature. Injury. 2016;47(3):559–67. 26830127 10.1016/j.injury.2016.01.007

[12] Heinanen M, Brinck T, Lefering R, Handolin L, Soderlund T. How to Validate Data Quality in a Trauma Registry? The Helsinki Trauma Registry Internal Audit. Scand J Surg. 2021;110(2):199–207. 31694457 10.1177/1457496919883961

[13] Curtis K, Gabbe B, Shaban RZ, Nahidi S, Pollard Am C, Vallmuur K, Priorities for trauma quality improvement and registry use in Australia and New Zealand. Injury. 2020 Jan;51(1):84–90. 31635906 10.1016/j.injury.2019.09.033

[14] Freeman D. What is the Keystroke-Level Model? [Internet]. 2022. Available from: https://info.keylimeinteractive.com/what-is-the-keystroke-level-model

[15] Palinkas LA, Horwitz SM, Green CA, Wisdom JP, Duan N, Hoagwood K. Purposeful sampling for qualitative data collection and analysis in mixed method implementation research. Adm Policy Ment Health. 2015 Sep;42(5):533–44. 24193818 10.1007/s10488-013-0528-y PMC4012002

[16] Kirchherr J, Charles K. Enhancing the sample diversity of snowball samples: Recommendations from a research project on anti-dam movements in Southeast Asia. PLoS ONE. 2018 Aug 22;13(8):e0201710. 30133457 10.1371/journal.pone.0201710 PMC6104950

[17] Simkis J. Simply Psychology. 2023. Snowball Sampling Method: Definition, Techniques & Examples. Available from: https://www.simplypsychology.org/snowball-sampling.html

[18] Saunders B, Sim J, Kingstone T, Baker S, Waterfield J, Bartlam B, Saturation in qualitative research: exploring its conceptualization and operationalization. Qual Quant. 2018;52(4):1893–907. 29937585 10.1007/s11135-017-0574-8 PMC5993836

[19] Nielson J. Nielsen Norman Group. 2012. Thinking Aloud: The #1 Usability Tool. Available from: https://www.nngroup.com/articles/thinking-aloud-the-1-usability-tool/

[20] Johnstone CJ, Bottsford-Miller NA, Thompson SJ. Using the Think Aloud Method (Cognitive Labs) To Evaluate Test Design for Students with Disabilities and English Language Learners. :25.

[21] Glaser BG. The Constant Comparative Method of Qualitative Analysis. Soc Probl. 1965;12(4):436– 5.

[22] Vindrola-Padros C. An introduction to rapid qualitative evaluation [Internet]. Social Research Association. [cited 2023 Sep 30]. Available from: https://the-sra.org.uk/SRA/SRA/Blog/Anintroductiontorapidqualitativeevaluation.aspx

[23] Nevedal AL, Reardon CM, Opra Widerquist MA, Jackson GL, Cutrona SL, White BS, Rapid versus traditional qualitative analysis using the Consolidated Framework for Implementation Research (CFIR). Implement Sci. 2021 Jul 2;16(1):67. 34215286 10.1186/s13012-021-01111-5 PMC8252308

[24] Lewinski AA, Crowley MJ, Miller C, Bosworth HB, Jackson GL, Steinhauser K, Applied Rapid Qualitative Analysis to Develop a Contextually Appropriate Intervention and Increase the Likelihood of Uptake. Med Care. 2021 Jun;59(6 Suppl 3):S242–51. 33976073 10.1097/MLR.0000000000001553 PMC8132894

[25] Goforth J. Center for Engaged Learning. 2023 [cited 2023 Oct 21]. Qualitative Methods for the Quantitatively Inclined. Available from: https://www.centerforengagedlearning.org/qualitative-methods-for-the-quantitatively-inclined/

[26] Crowe S, Cresswell K, Robertson A, Huby G, Avery A, Sheikh A. The case study approach. BMC Med Res Methodol. 2011 Jun 27;11(1):100. 21707982 10.1186/1471-2288-11-100 PMC3141799

